# Neurosurgical intervention in ultra-severe closed traumatic brain injury: Is it worth the effort?

**DOI:** 10.1016/j.bas.2024.102907

**Published:** 2024-08-13

**Authors:** Nikolaos Gkantsinikoudis, Iftakher Hossain, Niklas Marklund, Parmenion P. Tsitsopoulos

**Affiliations:** a Department of Neurosurgery, Hippokration General Hospital, Aristotle University School of Medicine, Thessaloniki, Greece; b Neurocenter, Department of Neurosurgery, Turku University Hospital, Turku, Finland; c Department of Clinical Neurosciences, Neurosurgery Unit, University of Cambridge, Addenbrooke's Hospital, Cambridge, United Kingdom; d Department of Clinical Sciences Lund, Neurosurgery, Lund University, and Skåne University Hospital Lund, Sweden

**Keywords:** Traumatic brain injury, Very severe, Ultra severe, Coma, Fixed pupils, Treatment, Decompression, Outcome, Glasgow coma scale 3-5

## Abstract

**Introduction:**

A subgroup of severe Traumatic Brain Injury (TBI) patients, known as ultra-severe (us-TBI), is most commonly defined as a post-resuscitation Glasgow Coma Scale (GCS) of 3–5. There is uncertainty on whether these critically injured patients can benefit from neurosurgical intervention.

**Research question:**

The available evidence regarding the decision-making and outcome following management of us-TBI patients is critically reviewed.

**Material and methods:**

Selected databases (PubMed, Google Scholar, Scopus and Cochrane Library) were searched from 1979 to May 2024 for publications on us-TBI patients, with a focus on treatment strategy, mortality and functional outcomes. Inclusion criteria were adult patients >18 years old with closed head trauma and admission post-resuscitation GCS 3–5. Studies were independently assessed for inclusion by two reviewers, and potential disagreements were solved by consensus.

**Results:**

Where such data could be extracted, mortality rate was 27–100%, and favorable outcome was observed in 4–30% of us-TBI patients. While early aggressive neurosurgical management was associated with decreased mortality, a high proportion of patients survived with unfavorable functional status.

**Discussion and conclusion:**

With supportive care only, outcome of patients with us-TBI is almost universally poor. Early and aggressive neurosurgical intervention in addition to best medical management can lead to favorable functional outcome in selected cases particularly in younger patients with an initial GCS>3 and traumatic mass lesions. There is insufficient data regarding the effectiveness of neurosurgical management on the outcome of us-TBI patients. and the decision to initiate treatment should be based on an individual basis.

## Highlights

• There is paucity of data on the outcome of patients with ultra-severe traumatic brain injury.
• Even with advances in neurocritical care and neurosurgery, outcome of ultra severe -TBI patients is frequently poor.
• Aggressive neurosurgical intervention can be considered in selected cases.
• Treatment options should be selected on an individual basis, considering numerous aspects during decision-making.

## 1 Introduction

Traumatic brain injury (TBI) represents a major cause of trauma-related death and disability worldwide, causing a substantial socio-economic burden to the society and the individual (O'Donnell et al., 2022). TBI severity is predominantly classified using the Glasgow Coma Scale (GCS) score on initial examination (Goswami et al., 2023). Severe-TBI (s-TBI) constitutes a distinct category of TBI, defined as an early GCS score of 3–8. These patients are frequently encountered in the acute trauma setting, presenting with a variable underlying intracranial pathology (Stocchetti et al., 2017; Van Dijck et al., 2018). Despite improvements in prehospital and neurocritical care management, morbidity and mortality in these patients remain high (Van Dijck et al., 2018; Frost et al., 2013; Carra et al., 2023; Lang et al., 2023; Wu et al., 2023).

Among s-TBI patients, there is a subgroup presenting with markedly impaired level of consciousness on initial assessment. These patients belong to a distinct category named “very-severe” or “ultra-severe” TBI (us-TBI), typically defined as those with an initial GCS score of 3–5 either before or after resuscitation (Van Dijck et al., 2018). Absence of pupil reactivity is also frequently encountered in this category, a finding that is used to guide treatment and it is considered an important prognostic factor for poor outcome (Tien et al., 2006; Tang et al., 2021; Tian et al., 2021).

Patients with us-TBI, GCS 3–5 and/or fixed and dilated pupils on admission represent a challenge regarding the decision to initiate aggressive neurosurgical and neurocritical care treatment, or to refrain from surgery in view of a likely dismal prognosis, regardless of maximal therapy. Hence, us-TBI patients who may benefit with survival and recovery to at least an acceptable functional status from acute therapy should be recognized to optimize and individualize acute management. Thus, clinical management and treatment decision in this subgroup of critically injured patients is challenging since clear guidelines are not available (Van Dijck et al., 2018). In view of the paucity of data guiding decision-making, there is limited evidence regarding neurosurgical management and associated neurologic outcome specifically targeting us-TBI patients. Interestingly, the most recent guidelines by the Brain Trauma Foundation (BTF) highlight the considerable variability during management of s-TBI patients and their outcome, without a focus on us-TBI patients (Carney et al., 2017; Robba et al., 2021). Further, in this category of patients, a number of ethical, financial and cultural issues are frequently encountered. Therefore, numerous factors should be carefully taken into consideration when selecting appropriate neurosurgical strategy (De Silva et al., 2009).

Since these patinets are frequently encountered in neurotrauma, a summary of the available evidence is needed to guide the clinician. This narrative review aims to critically summarize the available evidence regarding underlying pathology, main characteristics, neurosurgical management and clinical outcome of patients with us-TBI. Treatment controversies and overall prognosis, prognosis following maximal intensity neurosurgical treatment, ethical, cross-cultural considerations- and cost effectiveness of neurosurgical care for us-TBI patients are also discussed.

## 2 Literature review

For the purpose of this narrative review, selected databases (Pubmed, Google Scholar, Scopus and Cochrane Library) were searched from 1979, that is after the description of severe head injury based on the GCS score was initiated (Jennett et al., 1979), up to May 1st^,^ 2024 for publications on us-TBI patients. The following keywords were searched: “Trauma”, “Head injury”, “blunt trauma”, “coma”, “severe”, “fixed pupils”, “dilated pupils”, “fixed AND dilated pupils”, “intracranial hemorrhage”, “intracranial pressure monitoring”, “neurosurgical procedures”, “operative”, “decompression”, “hematoma removal”, “neurocritical care”, “osmotherapy”, “hyperventilation”, and “withdrawal of life support”. Furthermore, clinical trials such as DEcompressiveCRAniectomy (DECRA), Randomised Evaluation of Surgery with Craniectomy for Uncontrollable Elevation of Intracranial Pressure (RESCUEicp), Randomized Evaluation of Surgery with Craniectomy for Patients Undergoing Evacuation of Acute Subdural Hematoma (RESCUE-ASDH) as well as large epidemiological studies from registries such as Collaborative European NeuroTrauma Effectiveness Research in Traumatic Brain Injury (CENTER-TBI) and Transforming Research and Clinical Knowledge in Traumatic Brain Injury (TRACK-TBI) were reviewed (Cooper et al., 2011; Hutchinson et al., 2016; Kolias et al., 2022).

Inclusion criteria were adult patients with closed head trauma, and a post-resuscitation GCS score of 3–5. Moreover, studies including patients with bilateral fixed pupils were taken into account unless they referred to cases initially compatible with brain death. Papers not published in English language and studies with incomplete data regarding neurosurgical interventions, and outcomes were excluded. Two reviewers independently screened the relevant studies, extracted data and discussed disagreements until consensus was reached. In case of inconsistencies, the senior author (PPT) took the final decision.

**Fig. 1:**
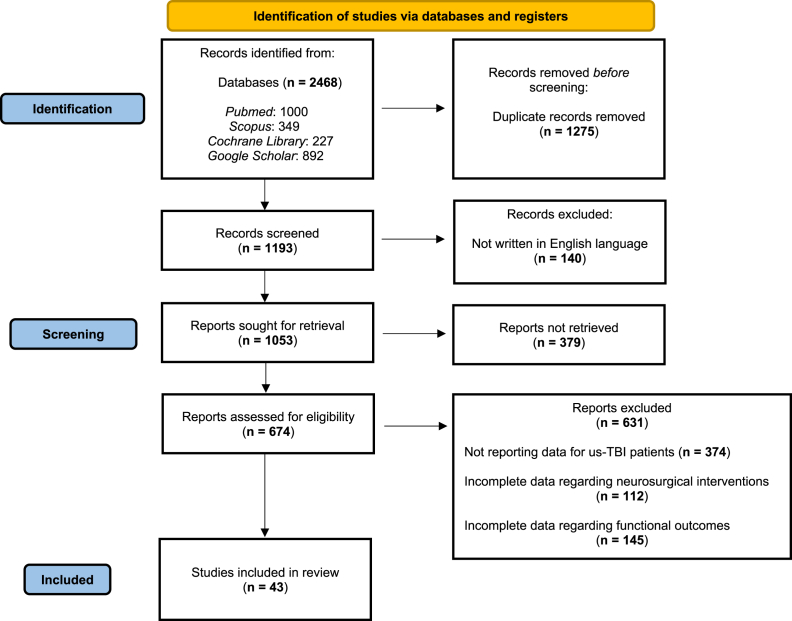
Flow Diagram showing the article selection process.

Initially, 2468 records were identified. Further screening resulted in 674 records that were checked for eligibility. Eventually, after applying specific criteria, 43 articles were eventually included in the analysis (Fig. 1).

## 3 Results

### 3.1 Neurosurgical intervention - mortality and functional outcome

As presented in Table 1, Table 2, Table 3, overall mortality varies greatly from 27.5 to 100%, and the rate of favorable outcome (Glasgow Outcome Scale, GOS, 4–5) from 0 to 29.7 %. It should be noted that important information in particular on pupillary abnormalities and time post-injury was often missing in retrieved studies (see Tables).

#### 3.1.1 Evacuation of mass lesion(s) (Table 1)

The effect of neurosurgical management of traumatic mass lesions in the setting of blunt us-TBI has been investigated in specific reports (Kawamata and Katayama, 2006; Chamoun et al., 2009; Chieregato et al., 2017; Salottolo et al., 2016; Lan et al., 2020; Griepp et al., 2023) (Table 1). All relevant studies were retrospective investigations, with varying sample sizes. Craniotomy with bone flap replacement or decompressive craniectomy (DC) with evacuation of underlying lesion was done. Individuals with us-TBI were encountered in 31.9–100% of enrolled patients among these studies, whereas initial pupil status was not always reported. Underlying lesion was extra-axial hematoma [epidural hematoma (EDH) or subdural hematoma (aSDH)], cerebral contusion or diffuse traumatic cerebral edema. Glasgow Outcome Scale (GOS) and overall survival, mortality and discharge rates were the primary outcome measures in the majority of the reports, principally at 6-month follow-up. Results have shown that mortality rates (GOS 1) ranged from 49.5 to 100%, whereas up to 14.6% of patients had a favorable outcome (GOS 4–5), percentages that correlate with age and admission pupil status. In an overall analysis, early and aggressive surgical intervention was associated with decreased mortality rates, and in some occasions a favorable functional outcome. Therefore, early recognition of these patients should be pursued but treatment decision must be individualized in view of the limited evidence (Kawamata and Katayama, 2006; Chamoun et al., 2009; Chieregato et al., 2017; Salottolo et al., 2016; Lan et al., 2020; Griepp et al., 2023)

**Table 1:** Overview of studies investigating the effect of craniotomy with bone flap replacement or decompressive craniectomy in patients with blunt us-TBI.

Author (Year)	Study Design	Sample Size (N)	Admission GCS scores (pupil status in us- TBI subgroup)	Underlying intracranial lesion	Interventions	Outcome Measures (follow-up period)	Outcome analysis	Conclusions
Kawamata and Katayama (2006)	Retrospective evaluation of surgical versus conservative management	182	GCS 9–15: 24.7% GCS 6–8: 43.4% GCS 3–5: 31.9% (N/A)	CC	Surgical excision of necrotic brain tissue with bone flap replacement (34%)	GOS (6 months)	Surgery was associated with decreased rates of poor outcome (GOS 1–3) in comparison with conservative management (92% versus 82%, respectively)	Benefit in us-TBI patients not specified
Chamoun et al. (2009)	Retrospective analysis of clinical outcomes of TBI patients with GCS: 3	189	GCS 3–5: 100 % (UFDP: 15.3%, BFDP: 36.5 %)	EDH, SDH, CC, Diffuse cerebral edema	Craniotomy and bone flap replacement (40.7%) DC and lesion management (7.9%)	GOS (6months)	Overall mortality rate 49.2%; 13.2% had a favorable functional outcome	Patients with GCS 3 should be aggressively treated in the setting of us-TBI, since favorable outcome can be achieved in a subset of patients
Salottolo et al. (2016)	Retrospective analysis of surgically treated us-TBI patients with GCS: 3 on admission	541	GCS 3–5: 100% (N/A)	SDH (58%) tSAH (53%) CC (40%)	Craniotomy and bone flap replacement (87%) DC and lesion removal (13%)	Mortality, survival and discharge rates	Survival rates were higher in the surgical group in comparison toconservative management group (61.2% vs. 50%, respectively) Functional outcome was similar in conservative and surgical groups	Neurosurgical management of GCS 3 patients may be associated with survival benefit in selected cases
Chieregato et al. (2017)	Retrospective analysis of medical management prior to craniotomy in patients with us-TBI and SDH	115	GCS 3–5: 100% (BFDP: 100%)	SDH (100%)	Craniotomy, SDH evacuation and bone flap replacement (11.3%) DC and SDH evacuation (88.7%)	GOS (12 months)	“Aggressive” hyperosmolar therapy (mannitol 1.0–1.5 g/kg) prior to surgery increased rates of favorable outcome (GOS 3–5), in comparison to basic” hyperosmolar therapy (mannitol <0.5 g/kg). (42.9% vs. 4.6%, respectively)	Aggressive medical management in us-TBI patients with BFDP and underlying SDH was associated with improved rates of favorable outcome
Lan et al. (2020)	Identification of DC prognosticators in patients with s-TBI (retrospective)	194	GCS 6–8: 46.9% GCS 3–5: 53.1% (UFDP: 73.2%, BFDP: 26.8%)	EDH (25.8%) SDH (45.4%) tICH (28.8%)	Craniotomy with bone flap replacement (26.3%) DC with underlying lesion management (73.7%)	GOS (6 months)	Mortality and unfavorable outcome (GOS 2–3) rates were high (48.5% and 36.9%, respectively)	DC can decrease mortality rates. Admission GCS was a strong prognosticator of final outcome
Griepp et al. (2023)	Retrospective analysis of patients with traumatic mass lesions, signs of brain herniation and non-reactive pupils after emergency surgical management	43	GCS 6–8: 23.3 of all patients GCS 3–5: 76.7% of all patients (UFDP: 25.6%, BFDP: 74.4%)	SDH (84.8%) EDH (9.1%)	Craniotomy and bone flap replacement (39.5%) DC and lesion management (60.5%)	mRS (12 months)	25.6% had a favorable functional outcome, 23.3% were severely disabled (mRS: 5)	Patients with transtentorial brain herniation, including those with bilaterally fixed and dilated pupils, may have higher chances of survival and functional recovery with aggressive medical and surgical management

* GCS: Glasgow Coma Scale; Us-TBI: Ultra-severe traumatic brain injury (admission post-resuscitation GCS 3–5); N/A: Not available; CC: Cerebral contusion; GOS: Glasgow outcome scale; ICP: Intracranial pressure; UFDP: Unilateral fixed dilated pupil; BFDP: Bilateral fixed dilated pupils; EDH: Epidural hematoma; SDH: Subdural hematoma; DC: Decompressive craniectomy; tSAH: Traumatic subarachnoid hemorrhage; s-TBI: Severe traumatic brain injury; tICH: Traumatic intracerebral hematoma; mRS: modified Rankin scale.

#### 3.1.2 Decompressive craniectomy (Table 2)

DC represents a common neurosurgical treatment in patients with us-TBI, enabling management of underlying traumatic lesions, and improving cerebral perfusion pressure with prevention of secondary brain injury. The role of DC in these patients has been investigated in a limited number of retrospective reports (Tang et al., 2021; Tian et al., 2021; Pompucci et al., 2007; Yuan et al., 2013) (Table 2). Admission rates of patients with us-TBI varied from 31.1 to 100%, whereas in two of these studies only patients with bilateral fixed dilated pupils were enrolled. The majority had unfavorable outcomes (GOS 1–3), with respective rates ranging from 70.3 to 91.5%, especially in patients with bilateral fixed dilated pupils on admission. However, other studies reported favorable outcomes (GOS 4–5), reaching up to one third of the patients (Yuan et al., 2013). Overall, the current evidence suggests that a subgroup of us-TBI patients could benefit from DC considering age, admission GCS and the time elapse from injury to surgery.

**Table 2:** Available evidence on outcome following Decompressive Craniectomy (DC) in blunt us-TBI patients.

Author (Year)	Study design	Sample Size (Ν)	Admission GCS scores (pupil status in us- TBI subgroup)	Underlying intracranial Lesion	Interventions	Outcome Measures (Follow-up period)	Outcome analysis	Conclusions
Pompucci et al. (2007)	Retrospective analysis of DC outcomes in TBI patients	55	GCS 9–15: 20.9% GCS 6–8: 22.7%GCS 3–5: 56.4% (N/A)	SDH and cerebral edema (62%) Diffuse cerebral edema (38%)	Unilateral FTP-DC (90.9%) Bilateral FTP-DC (9.1%)	GOS (12–102 months)	Us-TBI patients had higher rates of unfavorable outcome (GOS 1–3, 76.3%), when compared to GCS>5 patients	Mortality rates were high. A proportion of young patients had favorable outcomes
Yuan et al. (2013)	Retrospective analysis of DC with or without evacuation of underlying mass lesion in TBI patients	164	GCS 9–12: 37.2% GCS 6–8: 31.7% GCS 3–5: 31.1% (Abnormal UFDP or BFDP: 52.4%)	N/A	DC with mass evacuation (57.7%) DC without mass evacuation (42.3%)	GOS (2 months)	Good outcome (GOS 4–5) was obtained in 29.7% of us-TBI patients	DC and evacuation of underlying pathology resulted in lower mortality rates
Tang et al. (2021)	Retrospective analysis of DC outcomes in us-TBI patients with BFDP	94	GCS 3–5: 100% (BFDP: 100%)	SDH (86.2%) EDH (22.3% tSAH (91.5%)	Unilateral FTP-DC, Bilateral FTP-DC or Bifrontal DC (N/S)	Mortality (1 month) GOS (6 months)	Most patients had poor functional outcomes (89.4%, GOS 1–2)	Mortality and morbidity were high. Emergent DC should only be considered in young patients.
Tian et al. (2021)	Retrospective analysis of DC outcomes in s- TBI patients with BFDP	44	GCS 3–6: 100% (BFDP: 100%)	N/A	Unilateral FTP-DC (77.3%) Bilateral FTP-DC (22.7%)	Survival rates and GOS (Discharge, 6 and 12 months)	Unfavorable outcome (GOS 1–3) in 90.9% of patients	BFDP was associated with poor outcome

*GCS: Glasgow Coma Scale; Us-TBI: Ultra-severe traumatic brain injury (admission post-resuscitation GCS 3–5); N/A: Not available; SDH: Subdural hematoma; FTP-DC: Fronto-temporo-parietal decompressive craniectomy; GOS: Glasgow outcome scale; RCT: Randomized controlled trial; RESCUEicp: Randomised Evaluation of Surgery with Craniectomy for Uncontrollable Elevation of Intracranial Pressure; UFDP: Unilateral fixed dilated pupil; BFDP: Bilateral fixed dilated pupils; EDH: Epidural hematoma; tSAH: Traumatic subarachnoid hemorrhage.

### 3.2 Neurocritical care

#### 3.2.1 Intracranial pressure monitoring and medical management

In us-TBI patients where maximal treatment efforts are initiated, neurocritical management is essential either pre- or postoperatively, or both. ICP monitoring has been associated with decreased in-hospital and overall mortality rates in prospective investigations (Mauritz et al., 2008; Farahvar et al., 2012; Dawes et al., 2015). However, these results have not been fully verified by subsequent large studies, given the low compliance with BTF guidelines (Alice et al., 2017; Barami et al., 2021; Foote et al., 2022; Shim et al., 2023). Regarding multimodal neuromonitoring including brain tissue oxygen monitoring, recent large studies suggest that it is significantly associated with increased survival rates in s-TBI patients (Hoffman et al., 2021; Komisarow et al., 2022). Furthermore, implementation of conservative and medical measures such as osmotherapy, hyperventilation and barbiturates in a neurocritical care setting have been associated with increased rates of favorable outcomes in specific reports, despite the paucity of high-quality evidence regarding safety, efficacy and timing of interventions (Tian et al., 2021; Hutchinson et al., 2016; Kolias et al., 2022; Chamoun et al., 2009; Chieregato et al., 2017; Griepp et al., 2023; Pompucci et al., 2007; Yuan et al., 2013).

### 3.3 Prognostic factors of functional outcome

Independent of surgical intervention, there are specific non-modifiable factors that are strong prognosticators of functional outcome such as neurologic status on admission, unilateral or bilateral dilated and fixed pupils and age (Tang et al., 2021; Chieregato et al., 2017; Lan et al., 2020; Griepp et al., 2023; Pompucci et al., 2007; Yuan et al., 2013; Chaudhuri et al., 2009). It has been stated that TBI patients with GCS 3 and bilaterally fixed and dilated pupils have no possibility for survival (Chaudhuri et al., 2009). Further, an increasing number of studies enrolling elderly patients with us-TBI have emerged in the recent years (Tian et al., 2021; Hutchinson et al., 2016; Kolias et al., 2022; Salottolo et al., 2016; Pompucci et al., 2007). Indeed, aged patients have higher mortality rates and worse outcome, regardless of the performed intervention (Tang et al., 2021; Chamoun et al., 2009; Lan et al., 2020; Griepp et al., 2023; Pompucci et al., 2007; Yuan et al., 2013). Hence, age in conjunction with underlying comorbidities should be separately considered in decision-making and family counseling.

### 3.4 Withdrawal of life support

There is limited data on the withdrawal of life-supporting measures in patients with us-TBI. In a recent study, 37949 patients with severe TBI were included. Of them, 75.5% presented with GCS 3–4 and 11.1% with GCS 5–6. Withdrawal of life support was decided in 25.5% of those presenting with the lowest GCS score, and in 16.6% of those presenting with GCS scores of 5–6. In total, withdrawal of support was decided in 20.7% of whom 93% died while hospitalized. Age, lower GCS score, surgical decompression and higher injury severity score influenced this decision (Williamson et al., 2020).

Data from CENTER-TBI investigated the occurrence and timing of withdrawal of life support in patients with severe TBI. Of 2022 patients, ICU mortality was 13%. Of them, in 229 (11.3%) withdrawal of support was decided while 64.9% presented with motor GCS (mGCS) 1 and 7.3% with mGCS 2. Predicted probability for death and bad outcome was higher in those where early withdrawal of support was decided. Further, the most significant variables independently associated with early withdrawal of support were unresponsive pupils and injury severity score over 21 (van Veen et al., 2021).

### 3.5 Financial aspects

In Europe, about one-third of those hospitalized because of acute TBI, ca 700 000 cases annually, have sTBI (Majdan et al., 2016). Severe TBI, including us-TBI, is a major medical emergency, requiring high-level specialized care, treatment at ICU often for long periods, as well as long stay in hospital and rehabilitation. The mean treatment cost for the acute care of sTBI is > 16 000 € (Tuominen et al., 2012), but reaches about 130 000 € in those with the most severe injuries (Andelic et al., 2014). Although there is no available data exclusively on health economics for us-TBI patients, arguably the treatment costs of this patient group is substantial. This is more evident in resource-limited settings in low- and middle-income countries (LMICs), where continuing care of us-TBI patients in ICU for a long time might not be realistic (Allen et al., 2023). Even if the maximum therapy is possible in private hospitals, this might not be affordable for many families (Weiss et al., 2019). Thus, proper decision making for us-TBI patients not only has a significant impact on the quality-of-life of these patients, but also has a huge impact on the healthcare system globally since resources are frequently limited.

## 4 Discussion

The main message of this narrative review is that us-TBI is most often associated with high mortality and a high risk of poor functional outcome. However, mortality in some studies was as low as 27.5%, and the rate of us-TBI patients reaching a good functional recovery can be as high as 29.7%. Younger patients with GCS scores higher than 3 seem to have greater chances for better outcome. Nevertheless, most studies lack information of time post-injury, pupillary abnormalities and radiological criteria and overall, specific recommendations for initiating treatment cannot be provided.

Neurosurgical involvement including a variety of interventions in the acute trauma setting is crucial and can be life-saving in patients with severe TBI (Van Dijck et al., 2018; Rogers et al., 2011). Severe TBI patients presenting with very low GCS scores-the ultra-severe TBIs-may be considered candidates for surgical treatment, including those with bilateral dilated pupils with absence of pupillary light reflex. Traditionally, this observation in the setting of us-TBI was associated with uniformly poor prognosis (Tian et al., 2021; Chieregato et al., 2017). Nevertheless, a subgroup of us-TBI patients may reach a favorable outcome (Tang et al., 2021; Tian et al., 2021) (Table 3). The identification of patients with any potential for survival and an acceptable functional outcome remains a challenge.

**Table 3:** Summarized data (when available) on clinical outcome of us-TBI patients.

Study	Admission GCS scores (number of patients)	Age of us-TBI patients	Mortality (GOS 1)	Poor functional outcome (%) (GOS 2–3)	Favorable outcome (%) (GOS 4–5)
Kawamata and Katayama (2006)	GCS 9–15: 45 GCS 6–8: 79 GCS 3–5: 58	N/A	Surgery: 55 % Conservative: 70 %	Surgery: 27% Conservative: 22%	Surgery: 18% Conservative: 9%
Pompucci et al. (2007)	GCS 9–15: 12 GCS 6–8: 13 GCS 3–5: 31	N/A	76.3%		26.7%
Mauritz et al. (2008)	GCS 7–8: 467 GCS 5–6: 430 GCS 3–4: 959	N/A	51.1% in patients with GCS 3	N/A	N/A
Chamoun et al. (2009)	GCS 3–5: 189 (BFDP)	13–82 years	49.5% overall (In BFDP = 80%)	37.5%	13%
Farahvar et al. (2012)	GCS 6–8: 606 GCS 3–5: 761	N/A	N/A	N/A	N/A
Yuan et al. (2013)	GCS 9–12: 61 GCS 6–8: 52 GCS 3–5: 51	N/A	27.5%	42.8%	29.7%
Dawes et al. (2015)	GCS 4–6: 373 GCS 3: 449	N/A	N/A	N/A	N/A
Salottolo et al. (2016)	GCS 3–5: 541	49 ± 20 years (mean ± SD)	Not operated: 50.2% Operated: 38.8%	Ν/Α	Ν/Α
Chieregato et al. (2017)	GCS 3–5: 115 (BFDP), 62 not operated	34 years (median)	Not operated: 100% Operated: 75.5%	Not operated: 0% Operated: 20.5%	Not operated: 0% Operated: 4%
Piccinini et al. (2017)	GCS 6–8: 1328 GCS 3–5: 3352	N/A	N/A	N/A	N/A
Lan et al. (2020)	GCS 6–8: 91 GCS 4–5: 66 GCS 3: 37	Ν/Α	48.5%	36.9%	14.6%
Williamson et al. (2020)	GCS 7–8: 4578 GCS 5–6: 3499 GCS 3–4: 22003	Ν/Α	N/A	N/A	N/A
Barami et al. (2021)	GCS 6–8: 92 GCS 3–5: 107	N/A	74.1%	N/A	N/A
Hoffman et al. (2021)	GCS 6–8: 1673 GCS 3–5: 3335	N/A	N/A	N/A	N/A
Robba et al. (2021)	GCS 9–15: 339 GCS 6–8: 776 GCS 3–5: 1197	N/A	N/A	N/A	N/A
Tang et al. (2021) )	GCS 3–4: 94 (received DC)	2–82 years	78.7%	12.8%	8.5%
Tian et al. (2021)	GCS 3–6: 44 (BFDP)	14–82 years	63.6%	27.3%	9.1%
Foote et al. (2022)	GCS 6–8: 37 GCS 3–5: 84	Ν/Α	69.7% in patients with GCS 3	N/A	N/A
Komisarow et al. (2022)	GCS 3–5: 35501	40.3 years (mean)	33.4%	N/A	N/A

*Us-TBI: Ultra-severe traumatic brain injury (admission Glasgow Coma Scale 3–5 post-resuscitation); GOS: Glasgow Outcome Scale; N/A: Not available; BFDP: Bilateral fixed dilated pupils; aSDH: acute Subdural Hematoma; SD: Standard deviation; DC: Decompressive craniectomy.

**Reported mortality and functional outcome rates refer to us-TBI patients.

ICP monitoring is a cornerstone of neurocritical care management. Available evidence is insufficient to prove clear benefit of ICP monitoring or multimodality monitoring in the reduction of mortality or clinical improvement in patients with us-TBI. High-quality evidence is still lacking in this area (Carney et al., 2017; Moyer et al., 2023). On the other hand, the correlation between ICP levels and outcome in us-TBI shows substantial variation. Multimodal ICU monitoring in us-TBI patients may be advocated, since it may enable early recognition of disturbed intracranial physiology amenable for treatment (Lindblad et al., 2022; Lampros et al., 2023). However, any correlation with survival benefit and functional outcome has not been established and warrants further investigation.

A limited number of studies have assessed the value of surgical decompression (craniotomy or DC) for us-TBI (Tang et al., 2021; Chamoun et al., 2009; Chieregato et al., 2017; Salottolo et al., 2016). Besides clinical presentation, factors such as time from ictus to surgery, age, radiological findings and co-morbidities should be taken into consideration during decision making (Tang et al., 2021; Pompucci et al., 2007). Overall, early surgical decompression with craniotomy or DC in patients with us-TBI is associated with decreased early and late mortality rates. However, this increased survival usually comes at a cost of elevated rates of poor functional outcome among survivors (Tang et al., 2021; Tian et al., 2021; Hutchinson et al., 2016; Chieregato et al., 2017; Lan et al., 2020). A high proportion of patients may survive with persistent impaired level of consciousness and severe disability that includes substantial dependency for daily support. However, some younger patients with absence of comorbidities may benefit from surgical treatment, even those with preoperative signs of advanced brainstem compression (Tang et al., 2021; Pompucci et al., 2007). It should be mentioned though that clinical practices vary significantly among different centers and cultures, rendering comparative analyses between centers problematic (Gantner et al., 2022).

While prompt surgical decompression is a crucial part of treatment, the role of prehospital management that follows the Advanced Trauma Life Support principles must be also emphasized. Further, the contribution of ICP lowering measures such as hyperventilation, osmotherapy, hypothermia, sedation, analgesia and barbiturates may positively influence outcome (Hossain et al., 2023). With regards to surgery, acute surgical decompression is the most important treatment measure in patients with us-TBI. Although a primary DC is commonly applied, craniotomy and bone flap replacement is also an option, even in the presence of clinical and radiological signs of brain herniation (Chamoun et al., 2009; Chieregato et al., 2017; Salottolo et al., 2016; Lan et al., 2020; Griepp et al., 2023). To summarize, primary or secondary DC remains the mainstay of surgical treatment in us-TBI patients, since many of these patients have critical intracranial hypertension. Although DC is a standard procedure, there is increasing interest in potential modifications of the surgical technique in order to improve clinical outcomes (Jeong et al., 2020; Kumar et al., 2023).

Clinical management of us-TBI patients in the emergency setting is accompanied by remarkable ethical concerns. These are frequently difficult to discuss and manage with patients' relatives, given the paucity of existent evidence and guidelines as well as the minimal counseling time in the emergency setting. Cultural and religious beliefs must also be taken into consideration (Williamson et al., 2020). Family and next-of-kin should be thoroughly informed about high mortality and poor functional outcome rates in these patients, even with early and maximal treatment (Tien et al., 2006; Chaudhuri et al., 2009; Honeybul et al., 2013). The expectations from treatment should be outlined as soon as, and as realistic, as possible. Communication with family should take place often during hospitalization, ideally on a day-by-day basis (Rosenfeld and Mathiesen, 2023). Another factor that may remain unanswered is the patient's own will to survive or to terminate support in case of non-reversible substantial neurological deficits (Hutchinson et al., 2016; Rosenfeld and Mathiesen, 2023).

Available data and daily clinical practice indicate that injury severity, advanced age, and presence of comorbidities are strong driving forces for the decision to withdraw life support (Turgeon et al., 2011). The ideal timing to undertake this decision has yet to be determined, and should be individualized. However, in view of the consequences, delaying decisions beyond 72 h postinjury may prevent self-fulfilling prophecies (van Veen et al., 2021). The role of race, religion and geography on the decision to withdraw life support in us-TBI is complex and shows high heterogeneity (Diringer et al., 2001; Fiscella and Sanders, 2016).

The present review has specific method- and content-related limitations that may prevent generalization of results. Specifically, inclusion of involved articles was not conducted under a strict systematic review framework. In addition, a proportion of the analyzed data was retrieved from studies focusing on s-TBI patients although considerable effort was put to concentrate exclusively on us-TBI patients. Similarly, most published reports on neuromonitoring and surgical intervention in TBI patients focus on s-TBI patients, without specific data and subgroup analyses on us-TBI. As such, this narrative review could be an inspiration for future studies specifically targeting us-TBI patients. In addition, many reports do not precisely state pupillary and GCS status, radiological details such as midline shift or effacement of basal cisterns, timing from injury to surgical decompression and outcome in relation to lesion type. Furthermore, treatment, do not resuscitate (DNR) protocols, withdrawal of life support as well as decision-making for not initiating any treatment, represent crucial information that is consistently omitted.

## 5 Conclusions

Despite advances in neurocritical care and neurosurgical treatment options, management of us-TBI patients is challenging. Further, in spite of maximum efforts, outcome is frequently poor, with a favorable outcome observed in 4–30% of us-TBI patients. When dealing with us-TBI patients, the decision not to provide maximum therapy may pose a self-fulfilling prophecy. Nonetheless, aggressive neurosurgical intervention should be considered for the reduction of mortality and improved functional outcome in selected cases, followed by neurocritical care, in particular in younger individuals with localized hematomas presenting with GCS scores over 3. A reasonable approach is to provide maximal initial therapy unless clinical and radiological findings suggest imminent brain death. This would offer additional time for observation of the clinical and radiological course and provide time for counseling with family which is of crucial importance in order to meet realistic expectations.

The role of neurosurgery in us-TBI patients needs further investigation since the existent literature is insufficient. Until then, treatment options should be selected on an individual basis, considering available resources as well as clinical, radiological, ethical, and cultural aspects during decision-making.

## Declaration of competing interest

Not applicable. The authors declare that they have no known competing financial interests or personal relationships that could have appeared to influence the work reported in this paper.
